# Supplementary material for: Detection and Verification of QTL for Salinity Tolerance at Germination and Seedling Stages Using Wild Barley Introgression Lines
Source: Plants (Basel). 2021 Oct 21;10(11):2246. doi: 10.3390/plants10112246 (PMC8624391; doi:10.3390/plants10112246)
Supplement: Supplementary file 1 [file Reponses-for-all-reviewer-Plants.pdf › Reponses-for-all-reviewer-Plants.pdf]

Dear Editor in Chief, Plants

Dear Reviewers,

We would like to thank the editorial board of the **Plants** journal for their efforts. We would like to thank all reviewers for handling and thoroughly reviewing our manuscript. We appreciate the reviewers' critique, recommendations, and their constructive comments that greatly helped us improve the manuscript. We are happy to report that we were able to positively respond to all the reviewers' concerns. Please find our responses, all changes were made using Microsoft word track changes in the revised manuscript.

We look forward receiving a positive response!

Yours sincerely,

Sayed

The authors' responses to the reviewers

Reviewer: 1

Point 1:

1) ″ The authors used a set of wild barley introgression lines to detect the quantitative trait loci for salinity tolerance in barley. They focus on the phenotypes relating to seed germination and seedling development in response to salt treatment. Finally, they find the loci that might improve germination and seedling growth under salinity conditions. Both the idea and methods mentioned in the current paper are less of novelty. Several places in the methods are ambiguous.

Response 1: We thank reviewer 1 for the comments.

Selecting crop varieties with high tolerance to salt stress appears to be one of the most promising approaches to use the salt affected soil. Although some progress has been made to screen for salt tolerance for instance, imaging platforms using hyperspectral reflectance, the standard and conventional method of germination considers a suitable and widespread approach as its still used in laboratories to screen genotypes at the germination and seedling stages (Bliss et al. 1986) (Angessa et al. 2017). Screening genotypes at the germination and seedling stages in any crop are the most informative stages of the plant's lifecycle to evaluate the effect of salinity and determine seedling vigour.

In spite of barley a salt tolerance crop, few quantitative trait loci (QTL) and candidate genes associated with salt tolerance have been identified and reported. For this, the idea of our study targeting set of ILs tested for the first time for saline tolerance at germination and seedling stages as an initial stage in preparation for testing it under broader saline conditions in order to identify the genetic loci and alleles in this population to be useful source in breeding program as we could found interesting results, confirmed the presence of some genes or QTL loci associated with salt tolerance in the stage of adult plants grown in natural saline soil and irrigated with saline water as well in previous study (Sayed et al., 2021).

Point 2:

2) " Why do they choose the two concentrations of NaCl? ".

Response 2:

Sodium chloride (NaCl) is the most common salt in groundwater and soils and it's the common salt use in salinity experiment. We have chosen the two NaCl concentrations (75 and 150 mM) according to a preliminary experiment we did to evaluate ILs seed germination at different NaCl levels, we found that both selected level is more informative as they represent the medium and the high concentrations. Using higher NaCl concentration inhibit the germination of most of the genotypes. High concentrations of NaCl can reduce growth by the accumulation of high concentrations of both Na^+ and Cl^- simultaneously, but the effects of the two ions may differ. High Cl^- concentration reduces the photosynthetic capacity and quantum yield due to chlorophyll degradation which may result from a structural impact of high Cl^- concentration on PSII. High Na^+ interferes with K^+ and $Ca2^+$ nutrition and disturbs efficient stomatal regulation which results in a depression of photosynthesis and growth (as reviewed by Tavakkoli et al., (2010).

Point 3:

3) " At what stage of germination do they measure the shoot and root length?".

Response 3: The shoot and root lengths and seedling fresh weight were measured after ten days from germination, as mentioned in Materials and Methods section, subtitle salinity stress treatments, page 26, line 509 that the treatments were carried out in a versatile environmental test chamber for ten days. We could say these measurements were done at leaf development stage; first leaf through coleoptile (BBCH); first leaf unfolded (BBCH11) and two leaves unfolded (BBCH12) according to **BBCH-scale for cereals.** These stages varied from genotype to genotype based on their response to germination and growth under salinity treatments. In general, after ten days we measured seedling related traits.

Point 4:

4) " The unit of the germination time should be "hour" instead of "day".

Response 4: The germination time could be measured by day or hour. We think using "day " is more realistic to express how quickly germination is.as we followed Mudaris, (1984) and Ranal and De Santana (2006) who used "day " as a unit of mean germination time.

Point 5:

5) Units of the data in Table2 and Figure1 are missing.

Response 5):
Thanks for the hint. We added the full names, abbreviations and units below the Table 2 and Figure 1, page 5 lines 143-146 and page 7 lines 151-154.

Best regards, on behalf of all authors

Sayed

References:

Angessa, T. T., et al. "Early growth stages salinity stress tolerance in CM72 x Gairdner doubled haploid barley population." *PLoS One*, **2017** 12(6): e0179715.

Bliss, R. D., et al. (1986). "Osmotic sensitivity in relation to salt sensitivity in germinating barley seeds." *Plant, Cell and Environment*, **1986,** 9(9): 721-725.

Mudaris, M.A. Notes on Various Parameter s Recording the Speed of Seed Germination. *J. Agric. Trop. Subtrop.* **1984**, *99*, 147–154.

Ranal, M.A.; De Santana, D.G. How and why to measure the germination process? *Rev. Bras. Bot.* **2006**, *29*, 1–11, doi:10.1590/S0100-84042006000100002.

Sayed, M.A.; Nassar, S.M.; Moustafa, E.S.; Said, M.T.; Börner, A.; Hamada, A. Genetic Mapping Reveals Novel Exotic and Elite QTL Alleles for Salinity Tolerance in Barley. ***Agronomy*** **2021**, *11*, 1774, doi:10.3390/agronomy11091774

Tavakkoli, E.; Rengasamy, P.; McDonald, G.K. High concentrations of Na+ and Cl– ions in soil solution have simultaneous detrimental effects on growth of faba bean under salinity stress. *J. Exp. Bot.* **2010**, *61*, 4449, doi:10.1093/JXB/ERQ251.

Reviewer: 2

Point 1:

1) dentification of the salinity tolerance genes at early growth stages is an important subject to food production.

Response 1: Thank you for handling and thoroughly reviewing our manuscript. We totally agree with you that identifying genes at salinity-sensitive stages like germination and seedling development is of paramount importance in crop breeding programs for salt tolerance. Especially with the increasing areas of saline soils globally as a result of climatic changes and human practices.

Point 2:

2) In the introduction the mechanisms (as an example) should be described for surviving harsh environments apart from forming new genetic variations and alleles.

Response 2: As respond to the reviewer, we added seven sentences in the introduction part page 2 lines 44-51, discuss in brief the mechanisms of salinity on plants.

Point 3:

3) The material and methods should be simplified by showing specific activities and eliminating redundant descriptions, e.g. sentence 1.

Response 3: As respond to the reviewer, editing was done as much as possible and sentence 1 was adjusted.

Best regards, on behalf of all authors

Sayed

Reviewer: 3

Point 1: This manuscript is very well written. I only have two minor comments:

1) Figures 2 and 3 are difficult to interpret. This information could be better described in a table.

Response 1: Thank you for handling and thoroughly reviewing our manuscript. We tried to make the figures more clear, but unfortunately, they are still crowded so we have excluded them and d their data were described in Supplementary Tables S1 and S2, respectively.

Point 2:

2) In the "QTL detection" section in the Materials and Methods, please further describe the phenotype used as the response. Were LS means used as the phenotype?

Response 2: As respond to the reviewer, the Ls-means were used as the phenotype data following Honsodorf et al. (2017) and Naz et al. (2014).

Best regards, on behalf of all authors

Sayed

References:

Honsdorf, N.; March, T.J.; Pillen, K. QTL controlling grain filling under terminal drought stress in a set of wild barley introgression lines. *PLoS One* **2017**, *12*, 1–18, doi:10.1371/journal.pone.0185983.

Naz, A.A.; Arifuzzaman, M.; Muzammil, S.; Pillen, K.; Léon, J. Wild barley introgression lines revealed novel QTL alleles for root and related shoot traits in the cultivated barley (Hordeum vulgare L.). *BMC Genet. 2014 151* **2014**, *15*, 1–12, doi:10.1186/S12863-014-0107-6.

Reviewer: 4

In this manuscript, the authors evaluated phenotypic traits of a barley introgression line population and a recurrent parent under salinity treatments and then performed QTL analysis for those traits. The plant materials and methods were clearly described. However, I have several concerns as follows:

Point 1:

1) First of all, there were no line numbers in the manuscript, which made detailed comments very difficult. The authors should definitely include the line numbers in a revised version.

Response 1: Thank you for handling and thoroughly reviewing our manuscript. We apologize for this forgetfulness, and we have already numbered the lines in the revised version.

Point 2:

2) Secondly, although the design of this experiment is ok and the materials and methods section is concise and clear enough, the section of results was not well written/organized. The authors just simply considered all traits equally important and put all the results in the main manuscript. The results section should be better organized: the most interesting results should be emphasized and less important results should be put into the supplemental. It should not simply deposit all results in the main manuscript, but you need to really carefully think about the priority of them, and make your results description more interesting for readers.

Response 2: As respond to the reviewer, the results section was re-organized.

Point 3:

3) Thirdly, although the authors mentioned that some ILs were identified with good salinity tolerance performance and can be potentially used to develop new elite barley cultivars, the main objective of this study is to map QTLs for salinity tolerance traits. I cannot see from the manuscript how the identified QTLs can be used for future barley genetic studies, e.g. salinity tolerance gene cloning, or breeding practice for germplasm improvement, e.g., with marker-assisted selection or genomic prediction. I think that the usefulness of the detected QTL for future genetics/breeding studies is very important for this manuscript, and therefore, should be discussed. In addition, some salinity tolerance genes have been characterized in other crop species, e.g. GmCHX1 gene in soybean (Nat Commun 5, 4340), which might help for figuring out the most important traits in this study and make the manuscript more readable and interesting.

Response 3: We would like to thank reviewer 4 for his comment and we are totally agreed, we provided and discusses this point in page 26, lines 484-498.

Point 4: Detailed comments:

4) The whole section of results should be better organized and more concise, especially for those paragraphs and tables for QTL identification.

Response 4: As respond to the reviewer, the results section was re-organized.

Point 5:

5) All tables and figures should be better organized, and some less important results are not necessary to be displayed in the main text with a long table.

Response 5: As respond to the reviewer, the Figures 2 and 3 were excluded, and the data of them were described in Supplementary Tables S1 and S2, respectively. The important results were displayed in the main text and the long Table 5 was re-built.

Point 6:

6) In the section of Plant material and genotyping, better include the number of lines from the BC3S4 population, although it was mentioned in the abstract.

Response 6: As respond to the reviewer, the number of the BC_3S_4 population was included in page 25, line 620.

Point 7:

7) In the section of Statistical analyses, Pearson correlation analysis was not described. Tables 1-3: trait names should be identical among table 1-3 Figure 3: the barplots were too crowded to be visible. Should make each bar bigger and put them in supplemental. Why those traits generally had higher variation/noise than shoot length/root length shown in Figure 2?

Response 7: As respond to the reviewer, in the statistical analysis section Page 27, lines 565-566, we described the Pearson correlation coefficients. Trait names were unified in the whole manuscript. The Figures 2 and 3 were excluded, and the data of them were described in Supplementary Tables S1 and S2. These variations/noise might be due to the variations between replications of control treatments and those of 75 mM and 150 mM NaCl treatments. For example, the first replicate in the control treatment was tested against the first replicate in each of the 75 mM and 150 mM NaCl treatments, to calculate the STI. We calculated STI for each genotype in each replication in order to perform the ANOVA of STI.

Point 8:

8) Table 5: there are so many results to be displayed in the main text. Better select some most important results to display in the main text and put others in supplemental.

Response 8: As respond to the reviewer, we selected the most important results and remained in the Table 5 and the whole results were displayed in the supplementary Table S3.

Best regards, on behalf of all authors

Sayed
